# Granger Causality of the Electroencephalogram Reveals Abrupt Global Loss of Cortical Information Flow during Propofol-induced Loss of Responsiveness

**DOI:** 10.1097/ALN.0000000000003398

**Published:** 2020-07-01

**Authors:** Rebecca M. Pullon, Lucy Yan, Jamie W. Sleigh, Catherine E. Warnaby

**Affiliations:** 1 From the Department of Anaesthesiology, Faculty of Medical and Health Sciences, University of Auckland, Auckland, New Zealand (R.M.P., L.Y., J.W.S.); 2 the Wellcome Centre for Integrative Neuroimaging and Nuffield Division of Anaesthetics, Nuffield Department of Clinical Neurosciences, University of Oxford, Oxford, United Kingdom (C.E.W.).

## Abstract

It is a commonly held view that information flow between widely separated regions of the cerebral cortex is a necessary component in the generation of wakefulness (also termed “connected” consciousness). This study therefore hypothesized that loss of wakefulness caused by propofol anesthesia should be associated with loss of information flow, as estimated by the effective connectivity in the scalp electroencephalogram (EEG) signal.

**Methods:**

Effective connectivity during anesthesia was quantified by applying bivariate Granger to multichannel EEG data recorded from 16 adult subjects undergoing a slow induction of, and emergence from, anesthesia with intravenous propofol. During wakefulness they were conducting various auditory and motor tasks. Functional connectivity using EEG coherence was also estimated.

**Results:**

There was an abrupt, substantial, and global decrease in effective connectivity around the point of loss of responsiveness. Recovery of behavioral responsiveness was associated with a comparable recovery in information flow pattern (expressed as normalized values). The median (interquartile range) change was greatest in the delta frequency band: decreasing from 0.15 (0.21) 2 min before loss of behavioral response, to 0.06 (0.04) 2 min after loss of behavioral response (*P* < 0.001). Regional decreases in information flow were maximal in a posteromedial direction from lateral frontal and prefrontal regions (0.82 [0.24] 2 min before loss of responsiveness, decreasing to 0.17 [0.05] 2 min after), and least for information flow from posterior channels. The widespread decrease in bivariate Granger causality reflects loss of cortical coordination. The relationship between functional connectivity (coherence) and effective connectivity (Granger causality) was inconsistent.

**Conclusions:**

Propofol-induced unresponsiveness is marked by a global decrease in information flow, greatest from the lateral frontal and prefrontal brain regions in a posterior and medial direction. Loss of information flow may be a useful measure of connected consciousness.

## Abstract 2

In healthy adult volunteers, propofol anesthesia–induced loss of consciousness was associated with an abrupt, substantial, and global decrease in connectivity. These changes are comparably reversed at regain of consciousness. These observations suggest that information flow is an important indicator of wakefulness.

Supplemental Digital Content is available in the text.

## Visual Abstract:

**Figure:**
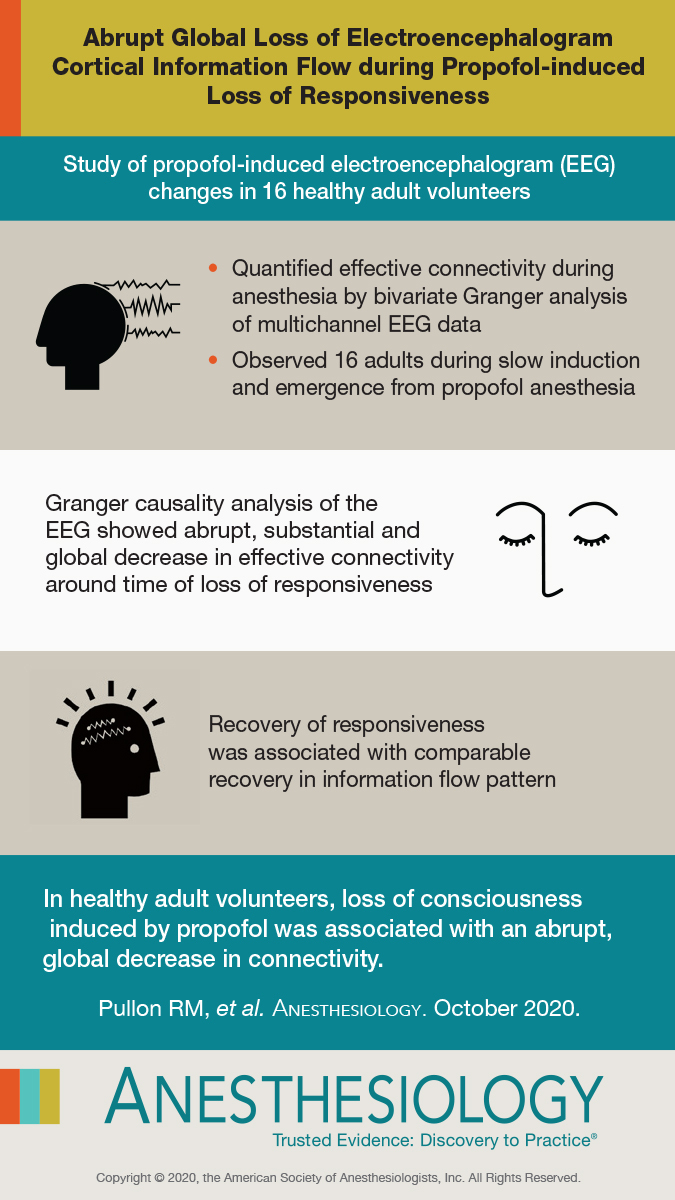


##

**Editor’s Perspective**

**What We Already Know about This Topic**

Information flow between brain regions is commonly hypothesized as a necessary component in the generation of wakefulness

The issue of how loss of consciousness alters this information flow is incompletely understood

Granger causality analysis of multichannel electroencephalogram recordings may provide a useful approach to study connectivity in the cerebral cortex

**What This Article Tells Us That Is New**

In healthy adult volunteers, propofol anesthesia–induced loss of consciousness was associated with an abrupt, substantial, and global decrease in connectivity

These changes are comparably reversed at regain of consciousness

These observations suggest that information flow is an important indicator of wakefulness

The use of electroencephalogram (EEG) and functional magnetic resonance imaging during anesthesia has enabled some progress in understanding the neural basis of consciousness. Yet the details of the transition to and from unconsciousness remain unclear. It is plausible that free information flux between regions allows the brain to generate the complexity associated with the conscious state, the so-called “connectionist” paradigm.^1,2^ If so, anesthetic-induced loss of consciousness should be accompanied by decreases in information measures. A number of studies have shown that general anesthesia might disrupt connectivity within and between large scale brain networks—as estimated by the EEG or functional magnetic resonance imaging—and this may be the mechanism for anesthetic-induced reduction in consciousness,^3^ because it is common to different classes of anesthetic drugs, sleep, and pathologic coma.^4^ In particular, the reduction in frontal-to-parietal interactions may be a key factor.^5,6^

A variety of different methods have been used to measure brain interactions, which has led to discordant results. Functional connectivity is estimated using measures of regional synchrony (coherence or correlation)—but, by definition, no information is flowing between perfectly synchronous sources. Because of differences in signal origin and temporal resolution, functional connectivity has been shown to decrease with anesthesia in functional magnetic resonance imaging studies,^7–9^ but to increase with anesthesia when applied to EEG data.^10,11^ Therefore, a number of directed measures have been developed to understand changes in regional brain information flux from the EEG signal (“directed functional” or “effective” connectivity). These quantify whether the information from a distant brain region has occurred just before that of the target region (directed phase lag index) and whether it has influenced the EEG time series at the target region (symbolic transfer entropy, Granger causality, dynamic causal modeling).^10,11^ In general, articles using symbolic transfer entropy and dynamic causal modeling align with the functional magnetic resonance imaging work, and show varying degrees of reduction in effective connectivity between brain regions with loss of responsiveness.^3,10^ The application of Granger causality is less consistent; some report anomalous increases in information flow with loss of responsiveness,^11,12^ while others found information flow to decrease with unresponsiveness.^11–13^ It is unclear whether these divergent results are real or are due to critical subtleties in the EEG signal processing.

Granger causality has been referred to as both directed functional connectivity^14^ and effective connectivity^15–17^; here we choose to describe Granger causality as an effective connectivity metric, to distinguish it from the synchrony measures typical of functional connectivity metrics. Therefore, in this article we test the hypothesis that propofol-induced loss and regain of responsiveness in healthy volunteers is associated with decreased information flow (effective connectivity) between regions of the brain using Granger causality of scalp EEG. We also compare the time course of this measure of information flow with that of regional synchrony using coherence (a functional connectivity metric). We focus on establishing the typical continuous trajectories of information flow during the transitions to and from unresponsiveness, and identify brain regions with the largest changes in information flow during these transitions.

## Materials and Methods

### Dataset

We reanalyzed a previously collected 31-channel EEG anesthesia dataset recorded from 16 healthy adult volunteers in Oxford, United Kingdom.^18^ The study was approved by the local Oxford University Research Ethics Committee, and written informed consent was given. The volunteers experienced a resting period with eyes closed and no drug administration for 10 min, followed by an ultraslow induction to loss of consciousness using propofol sedation. A target-controlled intravenous infusion of propofol was used with step increases of 0.2 μg/ml to achieve a maximum effect site concentration of 4 μg/ml over 48 min. After resting at the peak propofol dose for 10 min, the propofol sedation was switched off, and subjects were allowed to emerge to wakefulness while EEG recording continued for 48 min. Noxious laser, words, and tone stimuli were presented to the volunteers during the induction and emergence phases of the experiment. Time of loss and regain of behavioral responsiveness was assessed by button presses to an auditory cognitive word task approximately every 60 s. Volunteers were asked to respond using a two-option button box whether the two words presented were the same or different. Further details of the procedures and changes in EEG slow wave power are described in Ní Mhuircheartaigh *et al*.^18^ (“bench” dataset). The raw data is available at request *via* Zenodo (https://zenodo.org/record/1168447#.XuqPBC3Mync). No power calculation was conducted before the analysis presented in this article; sample size was based on the available data.

### Preprocessing of EEG

The EEG recordings were re-referenced to the average signal and downsampled to 125 Hz to ensure a reasonable model order for autoregressive modeling. For each electrode, a simple Hjorth-type spatial filter was applied by subtracting the average of the three surrounding electrodes. This is to mitigate the effect of noise, volume conduction, and global common mode signals and enhance the localization of EEG information.^19^ No other filtering was performed since filtering has been shown to disturb the information content and time ordering of data, which alters the regression coefficients of autoregressive models of the data and in turn interferes with the estimation of Granger causality.^20^ Artefacts were managed by excluding EEG segments with an amplitude above 200 µV.

### Effective Connectivity: Granger Causality

Granger causality describes the amount of information flow between two electrodes using autoregressive models, as described in the Supplemental Digital Content (http://links.lww.com/ALN/C400). We used frequency-domain Granger causality to quantify how information flow during propofol anesthesia changes with frequency. We opted to use bivariate (pairwise) Granger causality rather than multivariate Granger causality because we were interested in capturing the overall effects of propofol on the brain. Bivariate Granger causality is often criticized because it cannot distinguish between direct and indirect information flow between the source and the target, whereas multivariate Granger causality isolates the direct path between source and target by excluding all (known) common sources. The problem with multivariate Granger causality is that the exclusion of these common sources hides potentially important global effects. Propofol is acting to some degree on the whole brain, so it would be expected that a large proportion of the information flow would be mediated though indirect paths in the brain. If common effects are regressed out with multivariate Granger causality, it is likely that the global effects of propofol would be missed. Since we are interested in capturing the global effects of propofol, we therefore concentrated on bivariate Granger causality analysis. For the same reason, we have chosen not to do source-level analysis.

Granger causality was applied to nonoverlapping 4-s segments to each bivariate combination of electrodes for each subject. The EEG signals in each segment were first normalized by subtracting the mean and dividing by the SD. We chose short segment lengths of 4 s in order to balance the competing demands of stationarity (shorter time series are more likely to be stationary) and model fit (longer time series support better parameter estimation).^14^ This segment length is consistent with window lengths of 2 and 4 s that are commonly used for Granger causality analysis on EEG data.^13,14,21^ A model order of 3 (24 ms) was used for all Granger causality analysis. This model order was chosen as it minimized the Bayesian Information Criterion, calculated for 5,000 random windows from each subject for model orders 1 to 20. Akaike’s Information Criterion was also calculated to determine the optimal model order but often failed to produce a minimum. To test whether our results were robust to model order and sampling rate, we also ran our analysis on further downsampled data (62.5 Hz) and found no difference in the trajectory of Granger causality over time or in the electrode pairs that were subsequently identified to be dominant in the transition to and from unresponsiveness.

Morf’s modified Locally Weighted Regression method was used to calculate the autoregressive coefficients.^21^ The autocorrelation of the residuals was assessed with the Durbin–Watson test, and any EEG segments with *P* < 0.05 were excluded from the analysis. Frequency domain Granger causality was calculated at frequencies of 0.5 Hz and each whole number between 1 and 30 Hz. Thus, for each electrode pair in a subject’s EEG recording (930 unique pairs in total), we obtained a time-frequency Granger causality matrix (for example, see Supplemental Digital Content fig. S1, http://links.lww.com/ALN/C400). Granger causality trajectories over time for each electrode pair were smoothed by kernel regression^22^ using a Gaussian kernel with a bandwidth of 50 s. This form of nonparametric curve fitting avoids imposing an excessive degree of constraint on the resulting curves. Compared to a moving average, kernel regression produces a smoother trajectory while still capturing changes in direction.

### Functional Connectivity: Coherence

Coherence, a commonly used functional connectivity metric, was also calculated for each 4-s EEG segment. Coherence measures the alignment of phase angles between two electrodes. If the EEG signals from two electrodes are synchronous (*i.e.*, in phase), then coherence will be 1; if the signals are out of phase, then they will have a coherence of 0. Coherence is nondirectional, such that the coherence of electrode A to B will be the same as B to A, as coherence measures the synchrony between two signals rather than information flow. Coherence was calculated at the same frequencies as Granger causality: 0.5 Hz and whole numbers between 1 and 30 Hz. To extract the frequency of interest, the EEG signal was high- and low-pass filtered with third order Butterworth filters (0.8 Hz either side of the desired frequency), and instantaneous phases were computed with the Hilbert transform. Coherence is the mean difference in phase between two electrodes, weighted by power. Results were smoothed in the same way as Granger causality values, by kernel regression with a bandwidth of 50 s.

### Extracting Frequency-band Trends

We reduced the frequency dimension of the time-frequency spectrums of Granger causality and coherence by extracting the lower delta, alpha, and beta frequency bands. These frequencies are often of interest during the transition to and from consciousness. For each subject and metric, frequencies 0.5 and 1 Hz were averaged for the delta range, 8 to 14 Hz inclusive for the alpha range, and 21 to 30 Hz inclusive for the beta range. As the period around loss of behavioral response and recovery of behavioral response was of interest, the delta, alpha, and beta trajectories 15 min before and after each subject’s loss of behavioral response and recovery of behavioral response were extracted for each electrode pair. Granger causality values were normalized to the 95th centile value of the first 2 min of propofol infusion for that subject (*i.e.*, before any measurable brain concentrations of propofol were reached). This was considered a more appropriate baseline than the 10 min of resting before propofol infusion, because the subject’s eyes were closed during resting. A Granger causality greater than 1 therefore indicates a value larger than at the start of the propofol infusion. Normalizing in this way put Granger causality values for all subjects on a similar scale, allowing their trajectories to be combined appropriately. Coherence values were not normalized since they were already similar among subjects. The Granger causality trend for each electrode pair was summarized by the log-mean over all subjects, where loss of behavioral response and recovery of behavioral response were aligned as time zero. The log-mean was used since Granger causality is an asymmetrical, positive distribution and taking the logarithm transforms the data toward a normal distribution.^23^

### Identifying Networks and Regions of Interest

We were interested in identifying which brain regions showed large changes in Granger causality with change in level of consciousness, and also those that did not change much. Groups of electrode pairs with a large-magnitude Granger causality response at loss of behavioral response or recovery of behavioral response were identified by principal component analysis^24^ for each frequency band. The first principal component explained >90% of variability in the data for delta and alpha loss of behavioral response and recovery of behavioral response, so the electrode pairs in the top 5% of first principal component coefficients common to at least three of the four categories (delta loss of behavioral response/recovery of behavioral response and alpha loss of behavioral response/recovery of behavioral response) were selected to represent these large magnitude changes. This was repeated for the lowest 5% of first principal component coefficients to capture those electrode pairs with minimal change. Beta loss of behavioral response/recovery of behavioral response changes were excluded from the electrode pair selection because the magnitude of change was much smaller than for delta and alpha, and at these frequencies it is possible these changes are due to muscle activity (despite spatial filtering being used).

A similar principal component analysis was applied to the coherence loss of behavioral response and recovery of behavioral response trajectories; however, the trajectories were first divided into two groups according to whether coherence increased or decreased over the loss of behavioral response/recovery of behavioral response transition. Electrode pairs that were in the top 5% of first principal component coefficients for both loss of behavioral response and recovery of behavioral response transitions for each frequency band (*i.e.*, delta, alpha, beta) were selected.

### Statistical Analysis

Granger causality at various time points are presented as median and interquartile range of the mean bivariate trajectories over all participants. To facilitate visual presentation, some of the figures use logarithmic axes, and we also logarithmically transformed the data before modeling to achieve the normal distribution required. All statistical comparisons were two-tailed, with a significance level of 5%. Analysis was carried out in MATLAB 2019a (Mathworks, USA).

The groups of electrode pairs identified during the principal component analysis were analyzed across loss of behavioral response and recovery of behavioral response using a repeated measures ANOVA model. Granger causality was compared from 2 min before loss of behavioral response/recovery of behavioral response to 2 min after loss of behavioral response/recovery of behavioral response. Subject identifier and electrode pairs were included as between-subject factors, and time as a within-subject factor. Each frequency band and stage (*i.e.*, loss of behavioral response or recovery of behavioral response) was modeled separately.

We performed a further statistical analysis to ascertain whether the differences in Granger causality observed at the transition to unresponsiveness could be due to eyes closing rather than the anesthetic-induced changes in brain state. To separate out significant within- and between-subject effects, we fitted a linear mixed-effects model to logged Granger causality values at four time points for the electrode pairs identified to be in the top 5% of principal component analysis coefficients. The time points chosen to be representative of each stage in the recording process were as follows: 5 min (eyes closed, no propofol), 12 min (eyes open, propofol started), 60 min (eyes closed, subject unresponsive), and 110 min (eyes open, subject responsive). Fixed effects variables investigated were (i) eyes closed (true/false), (ii) propofol administered (true/false), and (iii) frequency band (categorical; delta, alpha, beta). The subject identifier number was included as a random effect.

## Results

Loss of behavioral response varied from 21 to 36 min after the start of the EEG recording (recall the propofol infusion started at 10 min after a period of resting) and recovery of behavioral response varied from 76 to 109 min. Two of the 16 subjects regained responsiveness less than 15 min before the end of the recording (at 7.5 and 12 min) and thus had incomplete data for 15 min postrecovery of behavioral response time segment. These datasets were still included in the analysis. A total of 886,538 four-second windows of EEG data were considered for analysis. A total of 4,384 (0.5%) EEG windows were excluded from the Granger causality analysis because of a maximum absolute amplitude of more than 200 µV. Since Granger causality acts on each bivariate pair of electrodes, the total number of Granger causality model fits was 26,464,620. The Durbin–Watson test excluded 892,189 (3.4%) of these fits due to residual autocorrelation. No exclusions were made for the coherence analysis. As previously reported, the EEG showed the usual increases in delta and alpha power with increasing propofol (Supplemental Digital Content fig. S2, http://links.lww.com/ALN/C400).

### Granger Causality: Overall Effects

**Fig. 1.**
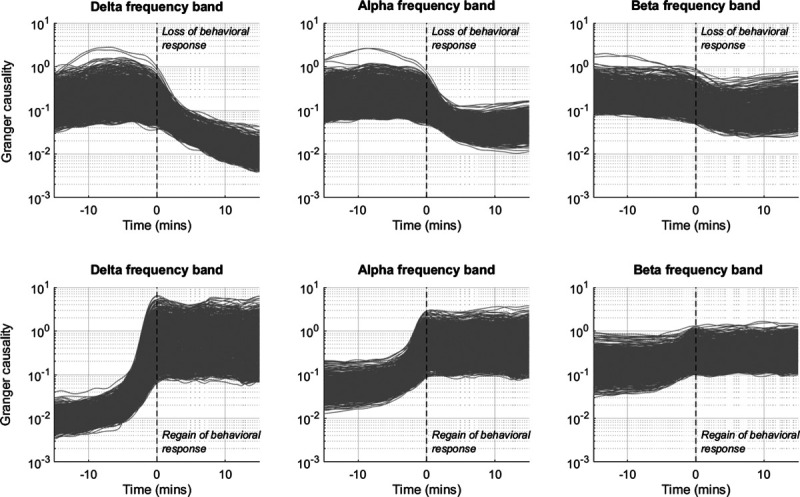
Mean Granger causality trajectories (expressed as normalized values) for the 15 min before and after loss of behavioral response (*top row*) and regain of behavioral response (*bottom row*) for the delta, alpha, and beta frequency bands. Each *gray line* represents one electrode pair (930 in total), calculated as the log-mean over all 16 participants. The *vertical black dashed lines* indicate the time of loss or regain of behavioral response. Note the logarithmic y-axis.

The normalized mean Granger causality trajectories for all electrode pairs around loss of behavioral response and recovery of behavioral response are shown in figure 1. The most obvious effect is that the state of unresponsiveness is marked by a profound decrease in information flow all over the brain. The largest magnitude changes are in the delta frequency band, where the median (interquartile range) of all the electrode pairs decreased from 0.15 (0.21) 2 min before loss of behavioral response (and 0.11 [0.12] at loss of behavioral response) to 0.06 (0.04) 2 min after loss of behavioral response (*P* < 0.001 *vs.* 2 min preloss of behavioral response); and 0.01 (0.005) 15 min after loss of behavioral response (*P* < 0.001 *vs.* 2 min preloss of behavioral response). Delta Granger causality returned to 0.48 (1.02) at recovery of behavioral response—that is, around four times the preloss of behavioral response level. This new Granger causality was sustained for 15 min postrecovery of behavioral response. The changes are least pronounced in the beta frequency range.

**Fig. 2.**
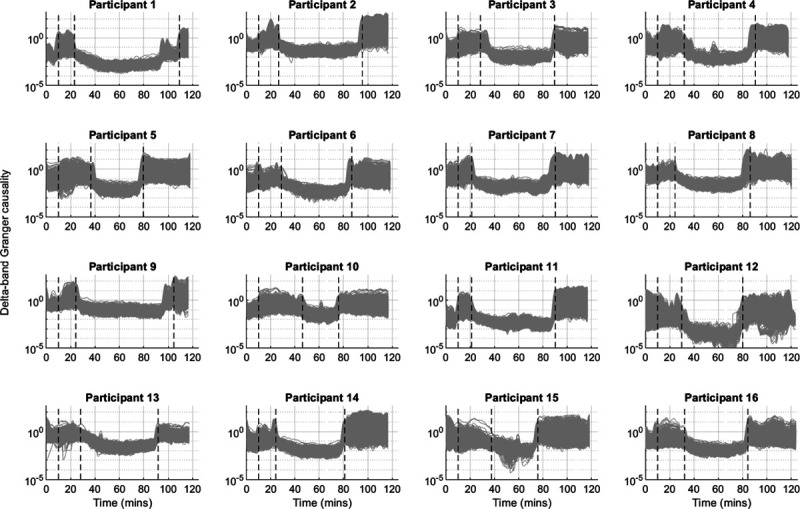
Delta-band Granger causality trajectories (expressed as normalized values) for individual participants. Each *gray line* represents the Granger causality trajectory of an electrode pair (930 in total). The three *vertical black dashed lines* indicate (i) start time of propofol (always at 10 min), (ii) time of loss of behavioral response, and (iii) time of regain of behavioral response. Note the logarithmic y-axis.

Individual participant trajectories across the entire time course are shown for the delta band in figure 2 (alpha and beta are included in Supplemental Digital Content figs. S3 and S4, http://links.lww.com/ALN/C400). It shows that, for 14 out of the 16 subjects, the decrease in Granger causality at the responsiveness transitions is very abrupt. This is suggestive of a process after that of an explosive (de)synchronization.^25^ We also see that for two subjects (*i.e.*, ID 1 and ID 9), there is a partial change in Granger causality just before recovery of behavioral response, followed by a bigger change at recovery of behavioral response—suggesting an initial failed return of responsiveness.

The mean of the absolute Granger causality residuals was found to be highly correlated with the mean Granger causality values (Supplemental Digital Content fig. S5, http://links.lww.com/ALN/C400); that is, as mean Granger causality increased, the variance around the mean also increased.

### Granger Causality: Regional Effects

**Fig. 3.**
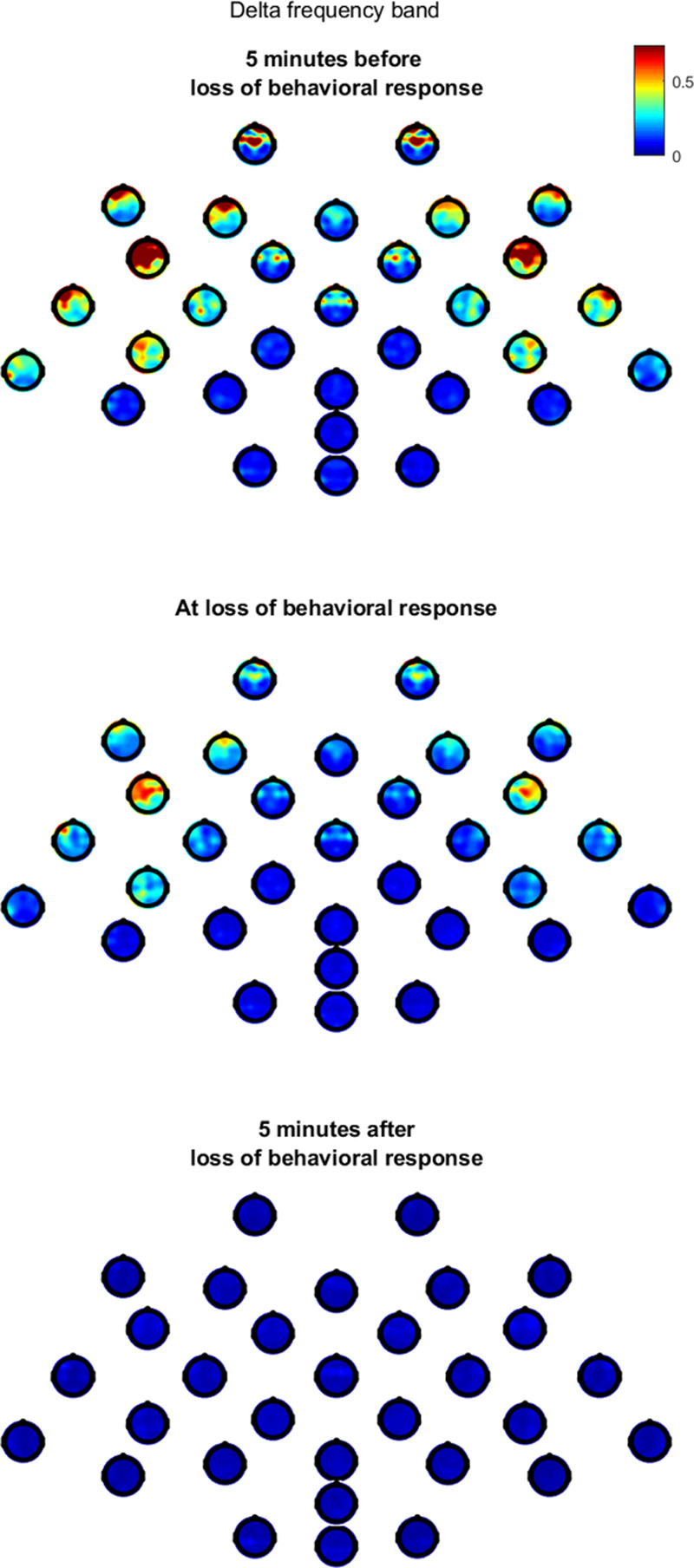
Two level hierarchical “topoplot-of-topoplots” for delta-band Granger causality (expressed as normalized values). For both the large composite topoplot and each small individual channel topoplot, the view is from the top of the head, oriented with the nose at the top and left hemisphere displayed on the left of the figure (*i.e.*, neurologic convention). The small topoplots at each electrode position shows the strength of the information flow (mean relative Granger causality) from that electrode to every other electrode for the delta frequency range. Three sets of topoplots are shown, with time relative to loss of behavioral response.

There was significant variability between different regions. Figure 3 presents a series of multiple topoplots that show the delta-band mean Granger causality value from each electrode to the rest of the head around loss of behavioral response. The largest changes in Granger causality are observed from lateral frontal and prefrontal electrodes in a posterior and medial direction. Information flow is roughly symmetrical across the left and right hemispheres at all timepoints. In contrast, the information flow from posterior to anterior regions is much smaller and appears unchanged around loss of behavioral response.

**Fig. 4.**
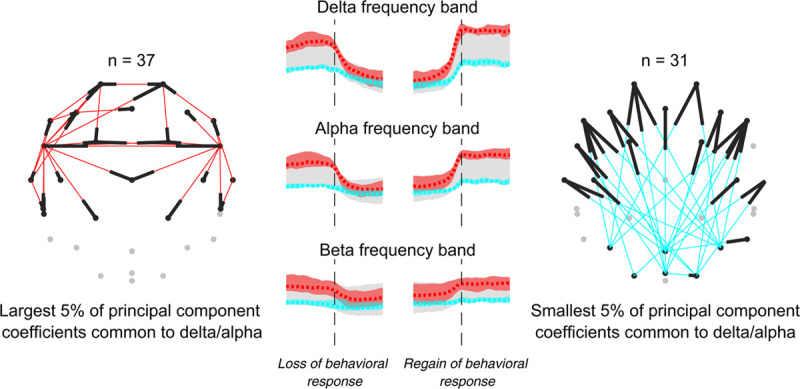
Visual representation of electrode pairs that have the largest 5% (*red, left*) and smallest 5% (*blue, right*) first principal component coefficients common to the delta and alpha frequency bands. That is, the electrode pairs with the largest and smallest changes in normalized Granger causality values during the loss and regain of behavioral responsiveness. Electrode pairs are illustrated on a headmap where the *black tips* of the connecting line indicate the destination electrode of the information flow (like the tip of an arrow, since Granger causality is directional). Each topoplot is a view from the top of the head, oriented with the nose at the top and left hemisphere displayed on the left of the figure (*i.e.*, neurologic convention). In the center, the median trajectory (*dashed line*) and the range (*shaded area*) of these electrode pairs are shown.

Principal component analysis of Granger causality trajectories returned 37 electrode pairs for the largest 5% of coefficients for delta and alpha loss of behavioral response/recovery of behavioral response and 31 electrode pairs for the smallest 5% coefficients (fig. 4). The position of the electrode pairs of these two groups in the principal component space is shown in Supplemental Digital Content figure S6 (http://links.lww.com/ALN/C400). The largest coefficients represent the electrode pairs with the greatest change in the 15-min window before and after loss of behavioral response/recovery of behavioral response. As evident in the topoplots in figure 3, these are predominantly from lateral electrodes to the medial (frontal, central, and parietal) regions, and from prefrontal to central electrodes. In the delta frequency band, the median (interquartile range) of this group of electrode pair means decreased from 0.82 (0.24) 2 min before loss of behavioral response to 0.17 (0.05) 2 min after. When taking into account electrode pair and participant, the changes across loss of behavioral response/recovery of behavioral response for all frequency bands were significant (repeated measures ANOVA, *P* < 0.001). Similarly, the smallest principal component coefficients identify those electrode pairs with minimal variability across loss of behavioral response/recovery of behavioral response. These are predominantly those associated with posterior to frontal information flow; however, even these showed statistically significant changes (*P* < 0.001).

The mixed effects modeling of the logged Granger causality demonstrated that both the eyes closed condition (*P* < 0.001) and propofol infusion (*P* < 0.001) had significantly lower Granger causality values across all electrode pairs. Thus, while closing of the eyes introduces modest reductions in Granger causality, it alone does not explain the total reduction in Granger causality during the transition to unresponsiveness, as shown by the following model equation:


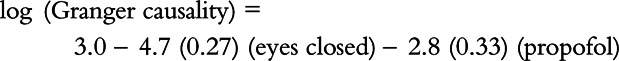


### Coherence

**Fig. 5.**
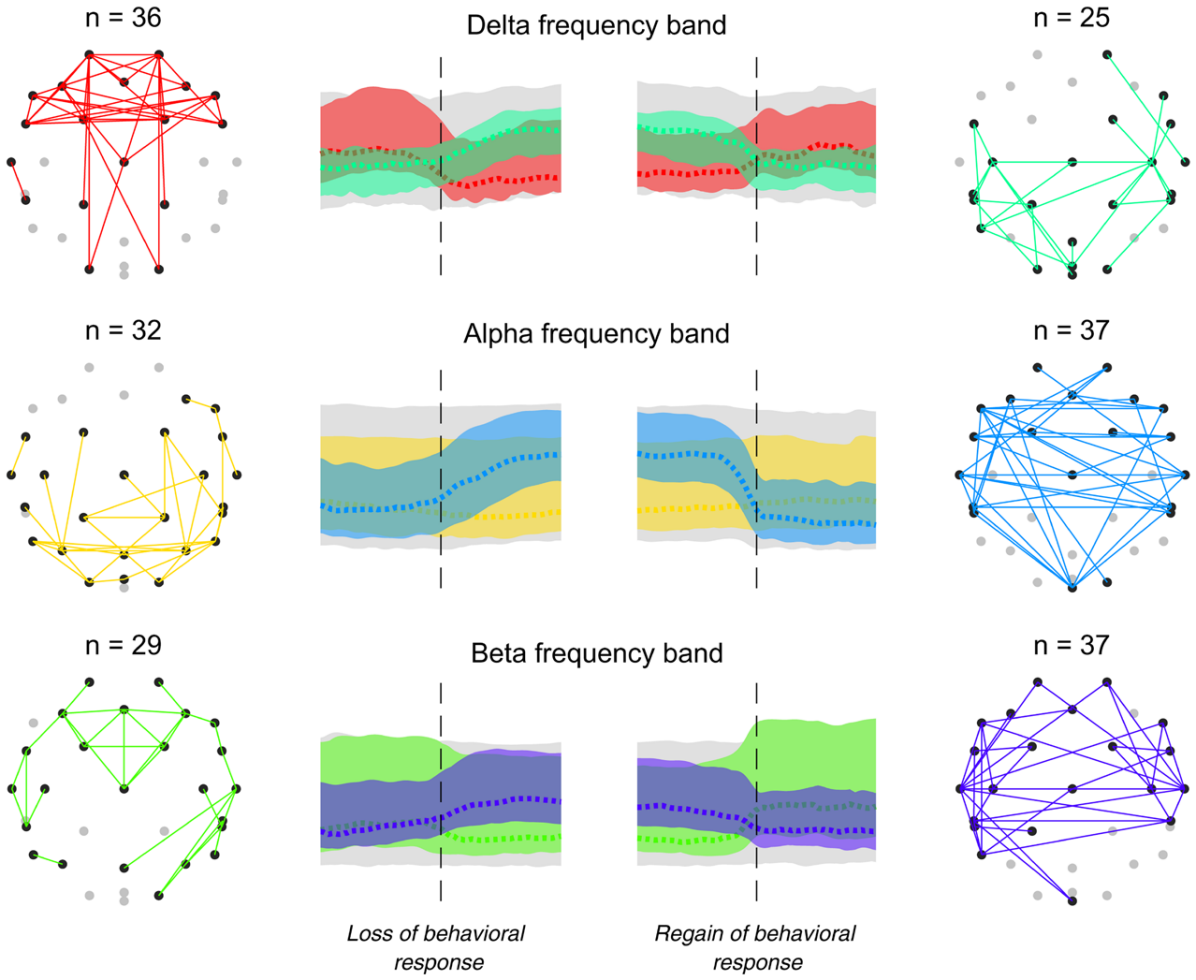
Visual representation of electrode pairs that have the largest 5% first principal component coefficients for coherence changes when considering those electrode pairs that decrease across loss of behavioral responsiveness (*left column*), and those electrode pairs that increase across loss of behavioral responsiveness (*right column*). The opposite trend typically occurs during regain of behavioral responsiveness. Principal components for the delta, alpha, and beta frequency bands are calculated independently. Electrode pairs are illustrated on a headmap in the outer columns, where the color of the connections corresponds to the colored trajectory across loss and regain of responsiveness (*inside columns*; *dashed line* indicates median, *shaded area* indicates range). Note coherence is nondirectional.

In contrast to the uniform decrease in Granger causality with propofol anesthesia, coherence decreased for some electrode pairs during anesthesia and increased for other electrode pairs, depending on the frequency (see Supplemental Digital Content fig. S7, http://links.lww.com/ALN/C400, for mean coherence trajectories for all electrode pairs). In general, delta band coherence decreased and alpha band coherence increased for the period of unresponsiveness. The electrode pairs identified by the principal component analysis on coherence are shown in figure 5. Decreases in delta band coherence are mainly among the frontal and prefrontal electrodes, with some connections to occipital electrodes O1 to O2: a median (interquartile range) decrease in electrode pair means from 0.31 (0.13) 2 min before loss of behavioral response to 0.25 (0.09) 2 min after loss of behavioral response (repeated measures ANOVA, *P* < 0.001). The increase in alpha band coherence is across hemispheres in frontal and central electrodes; a median (interquartile range) increase in electrode pair means from 0.24 (0.05) 2 min before loss of behavioral response to 0.29 (0.05) 2 min after loss of behavioral response (repeated measures ANOVA, *P* < 0.001). Beta band coherence also has some increase during anesthesia with a similar across-hemisphere pattern to alpha.

**Fig. 6.**
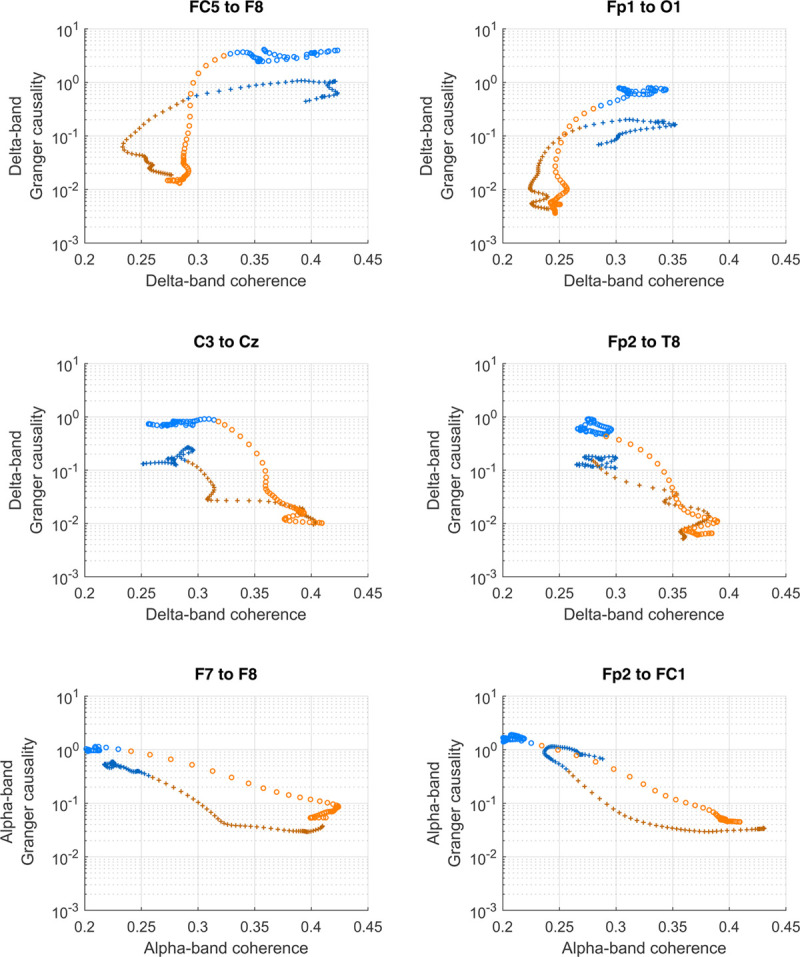
Comparison of Granger causality (expressed as normalized values) and coherence for six representative electrode pairs. The top row is two electrode pairs that have a decrease in delta coherence during anesthesia (FC5 to F8 and Fp1 to O1); the middle row is an increase in delta coherence (C3 to Cz and Fp2 to T8); and the bottom row is an increase in alpha coherence (F7 to F8 and Fp2 to FC1). Color indicates whether the measurements were during the responsive period (*blue*; before loss of responsiveness, after regain of responsiveness), or during the unresponsive period (*orange/red*; between loss of responsiveness and regain of responsiveness). “+” symbols are the loss of behavioral response transition; “o” symbols are the regain of behavioral response transition.

The groups of electrode pairs identified using Granger causality principal component analysis do not show consistent changes in coherence (Supplemental Digital Content fig. S8, http://links.lww.com/ALN/C400). Thus, it is apparent that coherence and Granger causality reflect different changes in the brain that occur with drug-induced unresponsiveness. Examples of the variability of state-space trajectories for coherence *versus* Granger causality for a select few electrodes are shown in figure 6. The 15 min before loss of behavioral response and after recovery of behavioral response (*i.e.*, responsive) are shown in blue, and the 15 min after loss of behavioral response and before recovery of behavioral response (*i.e.*, unresponsive) are shown in orange/red. The top row shows examples of electrodes where there is a decrease in coherence that precedes loss of behavioral response and the subsequent decrease in Granger causality. In contrast, the lower rows in figure 6 show examples of a negative correlation between Granger causality and coherence, *i.e.*, coherence increasing with anesthesia, but Granger causality decreasing.

## Discussion

We have presented the changes in Granger causality and coherence between 31 scalp electrodes during propofol-induced transitions to and from unresponsiveness. The most confronting result is the profound global decrease in information flow (*i.e.*, effective connectivity) between all electrode pairs for the duration of the unresponsive period. Granger causality drops to near zero at loss of behavioral response and is reversed at recovery of behavioral response. Although present at all frequencies, the Granger causality decrease is greatest in the delta frequency band and directed from lateral electrodes to the medial (frontal, central, and parietal) regions, and from prefrontal to central electrodes. In contrast, the changes in functional connectivity—as estimated by coherence—are less, and vary between different brain regions and frequency bands. The most marked effects are a decrease in delta band coherence in prefrontal-midline regions, and an increase in anterior cross-hemispheric alpha band coherence.

A global decrease in information flow during unresponsiveness has not previously been reported using the Granger causality metric. Barrett *et al.*^14^ reported a small increase in bidirectional Granger causality during loss of behavioral response (also in response to propofol) that was most pronounced in the beta and gamma frequency bands. However, there were some critical methodologic differences. They used a higher-order model (20; 80 ms) and examined only interactions between the midline structures (posterior and anterior cingulate cortices). Perhaps more important, their use of source localization would have the somewhat misleading effect of removing any global effects of anesthesia that were common to all electrodes. Bivariate Granger causality will estimate the total information flow between the two channels. In previous analyses, this has been seen to be a problem, because the “direct” information flow between the channels is almost always confounded by indirect information flow *via* other mediator linkages, or by a distant common source (with differing neuronal conduction delays). These are the so-called “latent confounders,”^26^ which have been blamed for introducing “spurious” correlations between brain sources. However, it is known that propofol acts on a wide range of brain targets—many outside the cerebral cortex. The use of multivariate Granger causality would therefore have the effect of being blind to any widespread effects of propofol, which may be the dominant cause of the anesthesia state. Such correlations could therefore be causal, not spurious. Using bivariate methods, we indeed found that propofol causes a widespread drop in total information flow between the EEG channels. This is in agreement with articles using symbolic transfer entropy^3,27^—which is a bivariate method of estimating information flow. In contrast, the articles by Barrett *et al*.^14^ (for anesthesia) and Lee *et al*.^28^ (for slow wave sleep) show either no effects, or a small increase in high-frequency direct information flow using multivariate Granger causality methods. We might therefore conclude that the decrease in cortical information flow is primarily mediated by propofol’s effects on distant circuits that coordinate the cortex, principally the thalamus, basal forebrain, and anatomical cortical networks.^29^ This is in complete agreement with the observation of chaotic traveling delta and slow waves seen with propofol anesthesia.^30^

The sudden switch-like change in Granger causality is seen clearly in the individual subject data, and seems to be more closely aligned with change in behavioral responsiveness than with the continuously and slowly changing propofol concentrations. Also, Granger causality did not progressively decrease much after the loss of behavioral response, suggesting that it is primarily an indicator of wakefulness—*i.e.*, conscious connectedness to the external world.^31^ Supportive of this is the fact that the electrodes that showed the biggest Granger causality decreases were overlying brain regions that reflect the functional anatomy required to perform the forced-choice auditory word task that was used to define behavioral unresponsiveness. The electrodes FC5/FC6 and F7/F8 estimate information flow from the extended Broca’s area (Brodmann Areas 47, 44, and 45), which is involved in language and semantic processing. Similarly, decreases in information flow from the orbitofrontal regions (Fp1-Fp2, Brodmann Area 10 to FC5/FC6 and Cz) could result in loss of executive function, and higher-order motor preparation required for the button presses. In contrast, the flow of information from the posterior/parietal brain areas is much smaller, and less affected around loss of behavioral response/recovery of behavioral response. Our findings are in keeping with previous work using symbolic transfer entropy, which showed a similar predominant decrease in lateral anterior-to-medial/posterior information flow with anesthesia.^3,5^ However, for the aforementioned reasons, we are cautious not to overinterpret local regional effects.

While the Granger causality always decreased with anesthesia, the coherence paradoxically increased with anesthesia in the central regions for the delta frequency band (fig. 6, middle row), and for most of the cortex in the alpha frequency band (fig. 6, bottom row)—a graphic example that increased synchrony may often be paradoxically associated with loss of information flow. Theoretically, information flow is minimal both with time series that are completely independent, and also with those that are perfectly correlated. Information flow peaks at an intermediate strength of causal linkage between the transmitter and receiver time series. Our results would suggest it is the information flow, rather than the regional synchrony, that is the important determinant of wakefulness.

### Granger Causality Considerations

Note that we follow convention in using the word “causality” solely in the context of Granger causality information flow. It could be argued that the term “Granger precedence” might more accurately reflect a somewhat limited Humean definition of causality as consisting only of temporal precedence,^32^ without including some physical influence/manipulation as a necessary component.

Although widely used, the application of Granger causality to neuroscience has engendered some controversy,^33,34^ because of both the somewhat abstract notion of information flow and its sensitivity to the details of its implementation. We have focused on the mean electrode-pair trajectories, averaged over the 16 subjects. We normalized each subject’s Granger causality values before combining and then took the mean of the logarithmically transformed Granger causality values (since the Granger causality distribution is chi-square rather than Gaussian). This method was effective for bringing out common trends among subjects, but its generalizability to individual subjects is unclear. We performed a sensitivity analysis by removing subjects with visually artefactual data (6/16), but there was no observed difference in the trajectory shape, so we included all subjects in our final analyses. Other measures of connectivity have shown more nuanced results when applied to clinical data^35^; we recommend this analysis is repeated on a larger, clinical dataset with high-density EEG systems.

#### Spatial Filtering.

We found muscle artefacts increased Granger causality values in the outer electrodes. This is because spurious causality appears when the electrodes are influenced by external sources that are not taken into account. The fact that these muscle artefacts were still present after taking the net Granger causality values would suggest differences in time lag from the external muscle sources. Thus we decided to use a spatial filter at the electrode level. A Laplacian filter would be the obvious choice, but as it is not recommended for less than 64 electrodes, we subtracted the mean of the surrounding three electrodes (Hjorth derivation). This is an approximate method that could spread large-amplitude artefacts, and may have an effect on the Granger causality values. However, we found no change in the overall trend of Granger causality during the transition to and from unresponsiveness with this spatial filter, and the Granger causality of electrode pairs with muscle-like artefacts was successfully diminished.

#### Stationarity.

Granger causality assumes stationary data. EEG windows of less than 10 s are generally assumed to be stationary, but this is not always true. In our data we found that variance often fluctuated within a data window. We removed windows where the EEG amplitude was above 200 µV to improve stationarity, but since we were not able to low-pass filter (due to the consequences on Granger causality estimation), about 0.1% of our windows remained nonstationary, and were excluded.

#### Bias.

Granger causality analysis in a sample yields an estimate that is biased away from zero, as compared to the “true” Granger causality value. Since we were more interested in the shape of the Granger causality trajectory during the transition to and from unresponsiveness rather than the absolute Granger causality values, we did not remove the bias from our estimates. Thus, we might have underestimated the true changes in Granger causality.

#### Nonlinear Information.

Granger causality is based on autoregressive modeling that captures linear information flow; nonlinear information flow is therefore not captured.

### Conclusions

At propofol-induced loss of responsiveness, cortical information flow—as assessed by bivariate Granger causality—abruptly decreases in all parts of the brain, although it is most pronounced in lateral, frontal, and central networks and in the delta frequency band. These changes are reversed at regain of responsiveness. The Granger causality results contrast with those of coherence—a nondirected measure of synchronous activity—suggesting that it is the information flow, rather than regional synchrony, that is the important indicator of wakefulness.

### Acknowledgments

The authors would like to thank the volunteers and team involved in data collection, and the reviewers for their helpful comments.

### Research Support

The analysis presented in this article is funded by a project grant (20/006) from the Australian and New Zealand College of Anaesthetists, and the James S. McDonnell Foundation (St. Louis, Missouri) grant No. 220023046. The original study was funded by the National Institute for Academic Anesthesia (United Kingdom) and the International Anesthesia Research Society. Dr. Warnaby is funded by the LUMINOUS Project and the Medical Research Council Development Pathway Funding Scheme (award ref. MR/R006423/1). The LUMINOUS project is the European Union’s Horizon 2020 research and innovation program H2020-FETOPEN-2014-2015-RIA under agreement No. 686764. The Wellcome Center for Integrative Neuroimaging receives core funding from the Wellcome Trust (United Kingdom; 203139/Z/ 16/Z). Funding also came from institutional and departmental sources.

### Competing Interests

The authors declare no competing interests.

## Supplementary Material


