# Supplementary material for: Granger Causality of the Electroencephalogram Reveals Abrupt Global Loss of Cortical Information Flow during Propofol-induced Loss of Responsiveness
Source: Anesthesiology. 2020 Jul 1;133(4):774–86. doi: 10.1097/ALN.0000000000003398 (PMC7495984; doi:10.1097/ALN.0000000000003398)

## SUPPLEMENTARY MATERIAL

Appendix 1: GRANGER CAUSALITY

Granger Causality describes the amount of information flow between two electrodes by using autoregressive models to assess whether past information in one electrode helps to predict current information in another electrode.[1-3] In this section, we present the theory of time-domain and frequency-domain Granger causality. For more information and computational implementation, we recommend Barnett and Seth.[4]

*Time-domain Granger causality*

For a time-varying signal $X_t$, a univariate autoregressive model can be used to predict the current value of its own signal based on its previous values. This is described by the following equation:

$$X_t = \sum_{n=1}^{k} a_n X_{t-n} + e_{xt} \qquad [1]$$

where $t$ is the current time point, $X_{t-n}$ are the values of the signal in the prior n time points, $a_n$are the corresponding autoregressive coefficients, and $k$ is the order of the autoregressive model (i.e. the number of previous time points taken into account). The error term, $e_{xt}$, is the difference between the true signal $X_t$and the predicted signal. If the model is a good fit to the data, the error will be small with small variability over time.

To capture whether the contribution of an additional time-varying signal, $Y_t$, can help predict the current value of $X_t$ the autoregressive model is extended to a bivariate model, which is described by the following equation:

$$X_t = \sum_{n=1}^{k} a_n X_{t-n} + \sum_{n=1}^{k} b_n Y_{t-n} + e_{xyt} \qquad [2]$$

where $Y_{t-n}$ are the values of the additional signal at the prior n time points, $a_n$ and $b_n$ are sets of autoregressive coefficients, and $e_{xyt}$ is the bivariate error term. In practice, $a_n$ and $b_n$ and hence $e_{xt}$ and $e_{xyt}$ can be derived by standard linear auto-regression methods, including ordinary least squares and multivariate Yule-Walker equations.

The Granger causality of signal $Y$ on signal $X$, $G_{xy}$, (i.e. the amount of information flow from electrode $Y$ to $X$) quantifies the degree to which the past of signal $Y$ helps to predict the current signal $X$; that is, it tests the null hypothesis that the coefficients $b_n$ are significantly different from zero. The log-likelihood ratio statistic for this test is the definition of Granger causality:

$$G_{xy} = \ln \frac{\mathrm{var}(e_x)}{\mathrm{var}(e_{xy})} \qquad [3]$$

If $Y$ does not contribute to $X$ then the errors and error variances for the univariate and bivariate cases will be the same (i.e. the coefficients $b_n$ will be zero so that the bivariate equation (Eq. 2)

reverts to the univariate case (Eq. 1)). In this case, since the natural logarithm of 1 is zero, $G_{xy}$ will be zero. As the bivariate error term decreases (because previous values of $Y$ help predict current values of $X$), the error variance ratio increases, and hence so does $G_{xy}$. Thus, greater values of $G_{xy}$ indicate a larger amount of information flow from electrode $Y$ to $X$. For example, Figure A illustrates the contribution of $X$ and $Y$ to the predicted $X$ signal for a low and high Granger causality case.

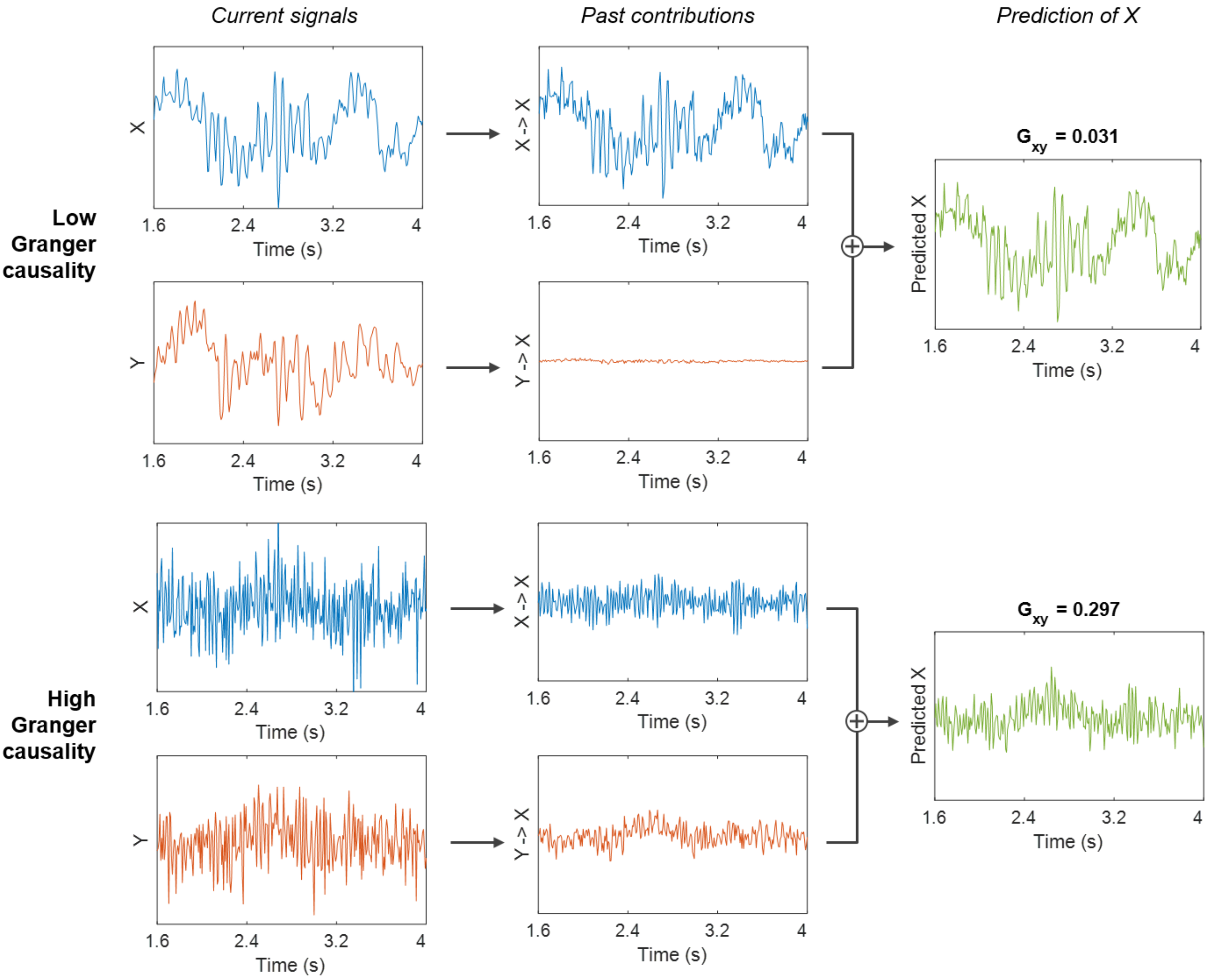


**Figure A:** Granger causality assesses how much of the past signal $Y$ contributes to the prediction of the current signal $X$. On the left, two sets of $X$ and $Y$ EEG signals are shown. Their relative contributions towards predicting the future $X$ signal are shown in the middle. In the top example, $Y$ does not contribute much to the future $X$ signal illustrated by the low Granger causality value of 0.031. In contrast, the bottom example shows that $Y$ reduces the error of the predicted $X$, with a Granger causality value of 0.297.

### *Frequency-domain Granger causality*

Granger causality can also be evaluated in the frequency domain, where spectral decomposition is used to restrict inferences about information flow to particular frequency bands. Spectral Granger causality can be thought of as measuring the proportion of power of $X$ at the given frequency that is derived from its interaction with $Y$. In comparison, time-domain Granger causality as described above can be considered to be an average over all frequencies of the spectral region.

The time varying $X_t$ and $Y_t$ signals have associated power spectral density functions $S_{xx}(\lambda)$ and $S_{yy}(\lambda)$, where $0 \leq \lambda \leq 2\pi$. The cross-power spectral density, $S$, is defined as the (two-sided) Fourier transform of the autocovariance sequence. However, for autoregressive models there is a unique spectral factorisation for $S$ with a transfer function, $H$:

$$S(\lambda) = H(\lambda)\,\Sigma H(\lambda)^* \quad [4]$$

where * denotes the inverse and $\Sigma$ is a matrix of the error variances from the autoregressive model. The transfer function is the inverse matrix of the Fourier transform of the regression coefficients:

$$H(\lambda) = \left(I - \sum_{k=1}^{p} A_k e^{-ik\lambda}\right)^{-1} \quad [5]$$

For the bivariate autoregressive model (of signals $X$ and $Y$), $S$ and $H$ are 2x2 matrices, such that:

$$S(\lambda) = \begin{bmatrix} S_{xx}(\lambda) & S_{xy}(\lambda) \\ S_{yx}(\lambda) & S_{yy}(\lambda) \end{bmatrix} \quad [6]$$

The spectral Granger causality from $Y$ to $X$ is therefore defined as:

$$G_{xy}(\lambda) = \ln\left(\frac{|S_{xx}(\lambda)|}{|S_{xx}(\lambda) - H_{xy}(\lambda)\Sigma_{yy}H_{xy}(\lambda)^*|}\right) \quad [7]$$

All Granger causality analysis in this paper is at a specific frequency between 0.5 and 30 Hz, calculated using this frequency-domain representation.

*References*


1. Geweke J: Measurement of linear dependence and feedback between multiple time series. *J Am Stat Assoc* 1982; 77: 304-313
2. Granger CW: Investigating causal relations by econometric models and cross-spectral methods. *Econometrica* 1969: 424-438
3. Cohen MX: Analyzing neural time series data: theory and practice, MIT press, 2014
4. Barnett L, Seth AK: The MVGC multivariate Granger causality toolbox: a new approach to Granger-causal inference. *J Neurosci Methods* 2014; 223: 50-68

Figure S1:

Time-frequency spectrum for Granger causality on a log scale. This is from participant 1, bivariate electrode pair F7 to Fz (i.e. front left to front central).

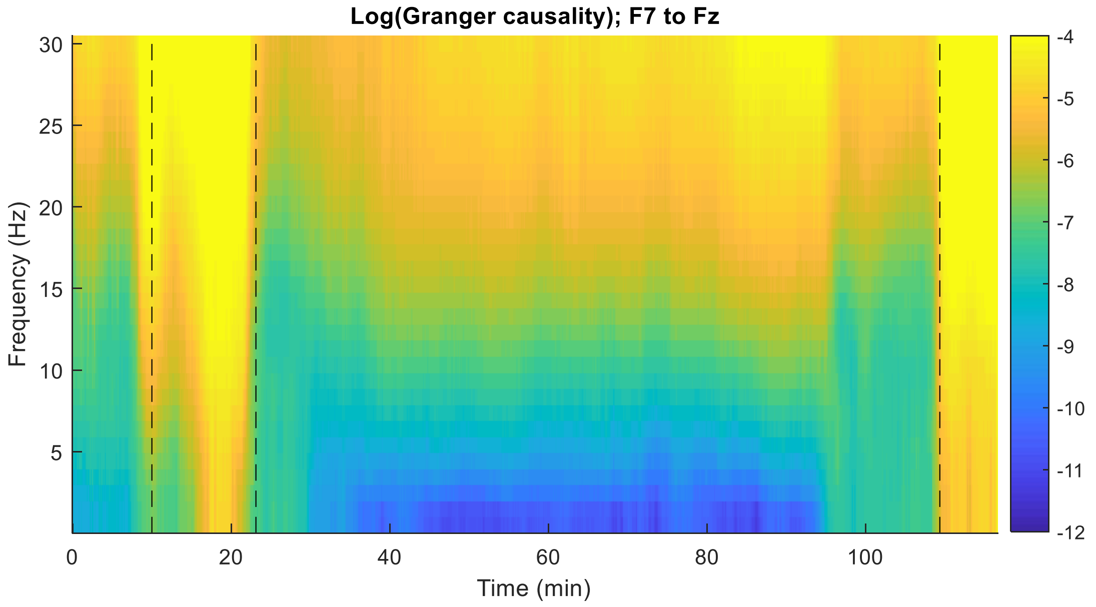


Figure S2:

Power trajectories of the delta, alpha, and beta frequency bands for the 15 minutes either side of loss of behavioral response (top row) and regain of behavioral response (bottom row).

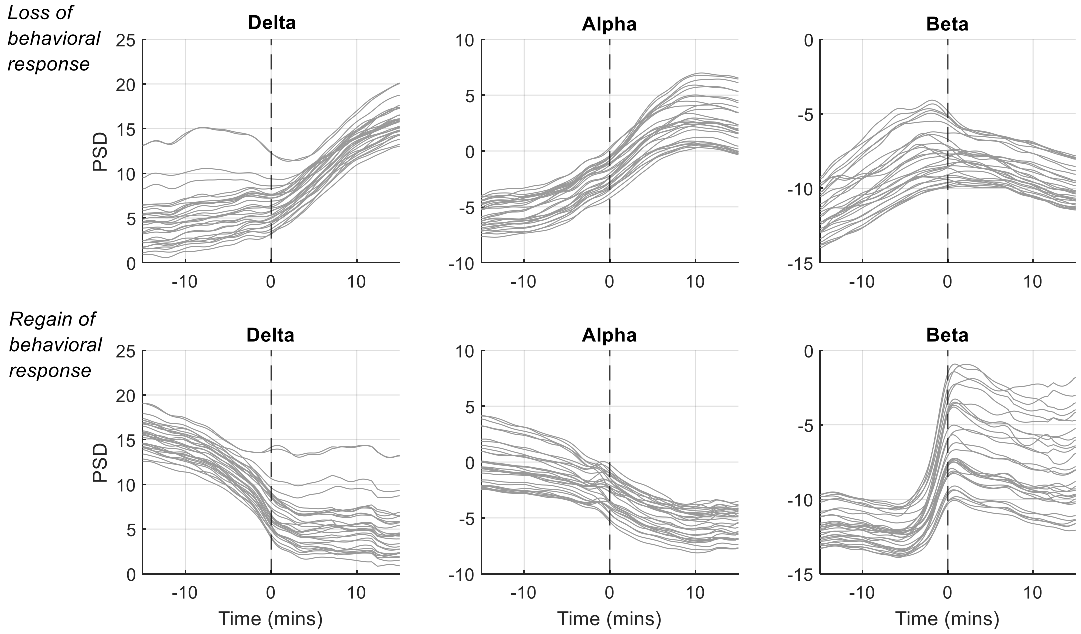

Figure S3:

Alpha (8-14Hz) Granger causality trajectories by subject (normalized values shown). The three vertical dashed lines indicate: (i) the start of propofol (always at 10 minutes), (ii) time of loss of behavioral response, and (iii) time of regain of behavioral response.

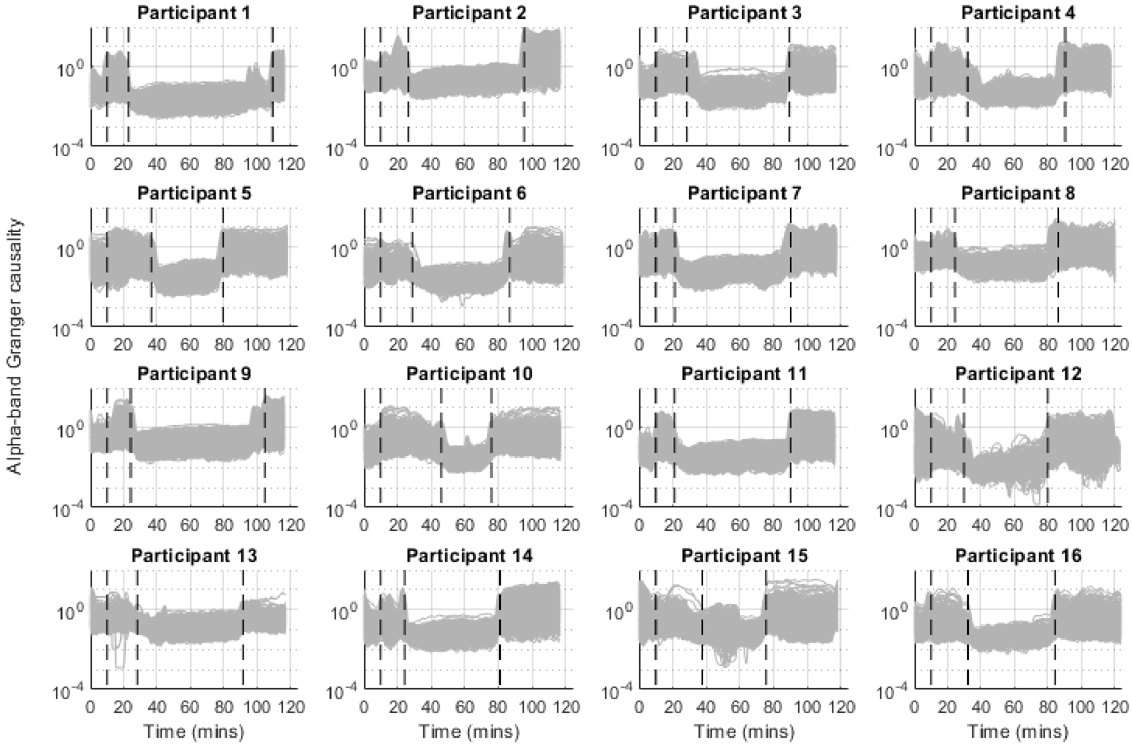


Figure S4:

Beta (21-30Hz) Granger causality trajectories by subject (normalized values shown).

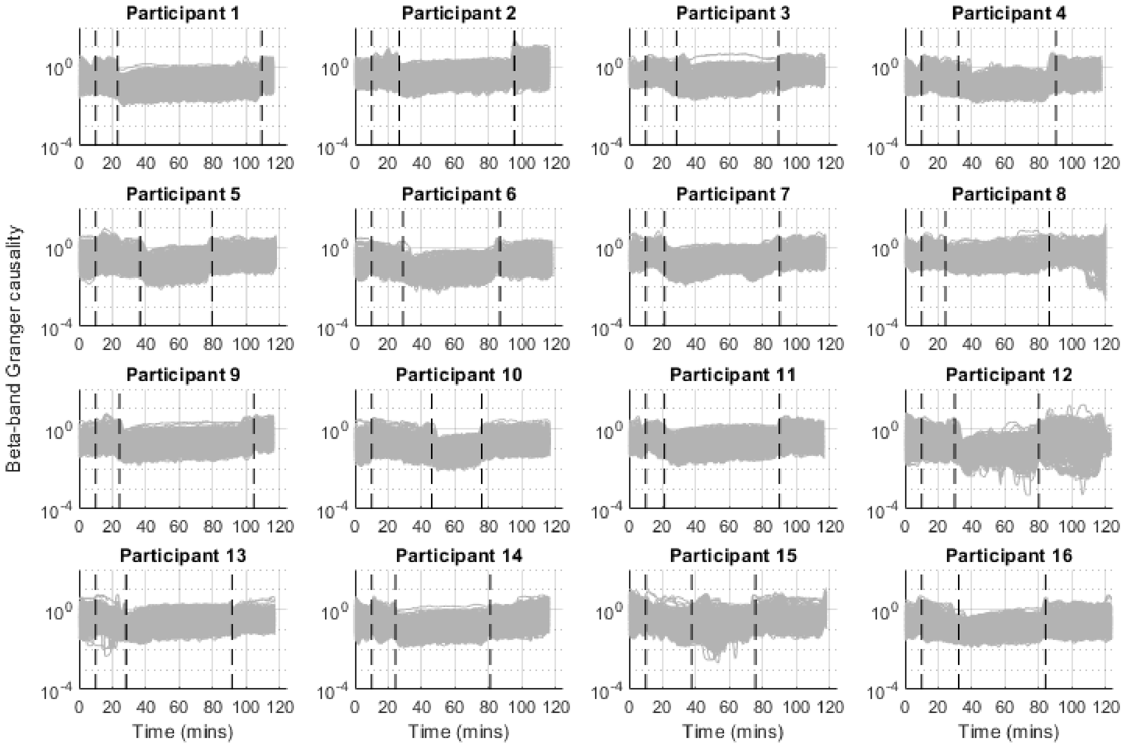

Figure S5:

Investigation of Granger causality residuals. For selected electrode pairs, Granger causality values (normalised, 'GC') for the delta frequency band are plotted against their residuals (also normalised, 'Resid') for the 15 minutes either side of loss of responsiveness.

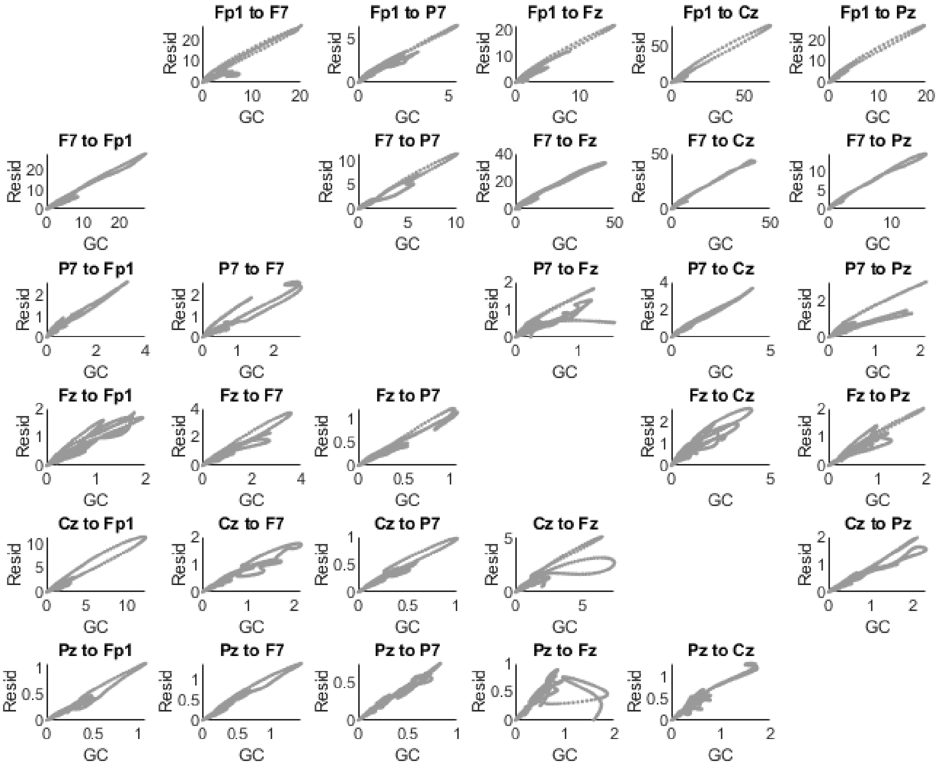


Figure S6:

Principal component analysis on Granger causality values, shown in the first two dimensions of the principal component space (PC1 = first principal component; PC2 = second principal component). The electrode pairs with coefficients in the top 5% are highlighted in red; the lowest 5% are in blue. These correspond to the colors in Figure 4 of the main paper.

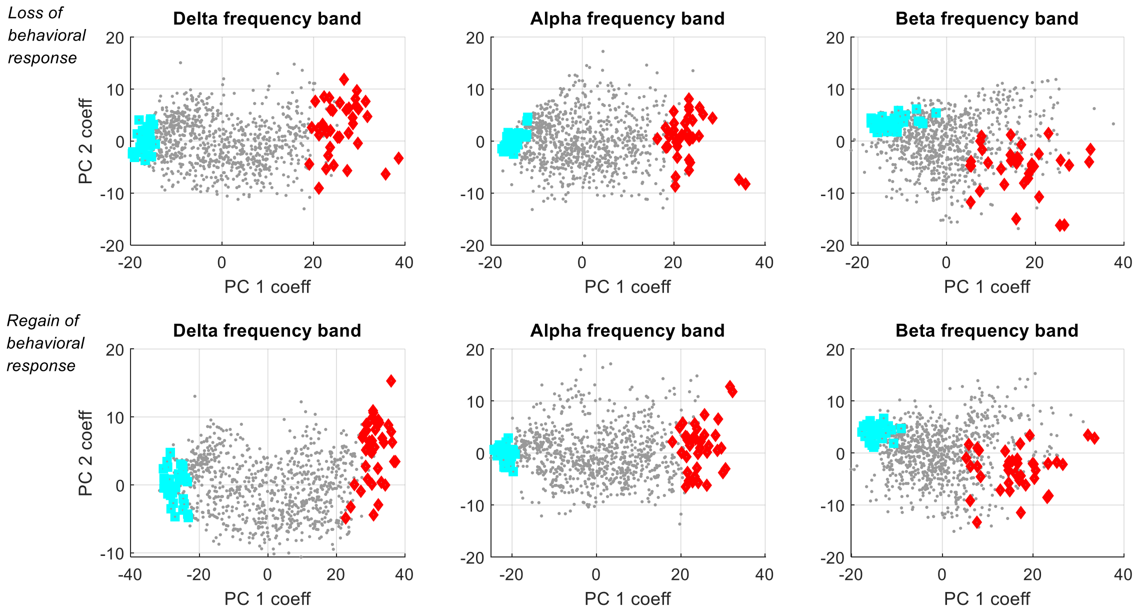

Figure S7:

Coherence trajectories of each electrode pair (median over all participants) for the 15 minutes before and after loss of behavioral responsiveness (top row) and regain of behavioral responsiveness (bottom row). The delta, alpha and beta frequency bands are shown separately. Note the y-axis is logged.

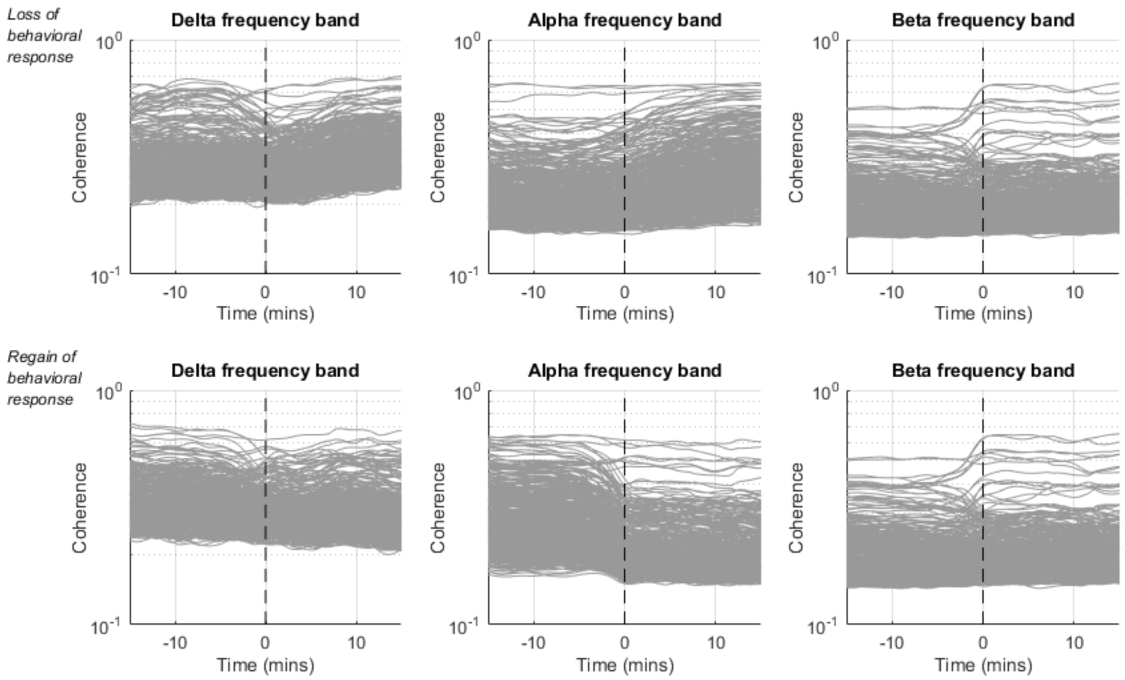


Figure S8:

The electrode pairs identified during the Granger causality principal component analysis (i.e. the top 5% and lowest 5% principal component coefficients) are shown here for the coherence trajectories.

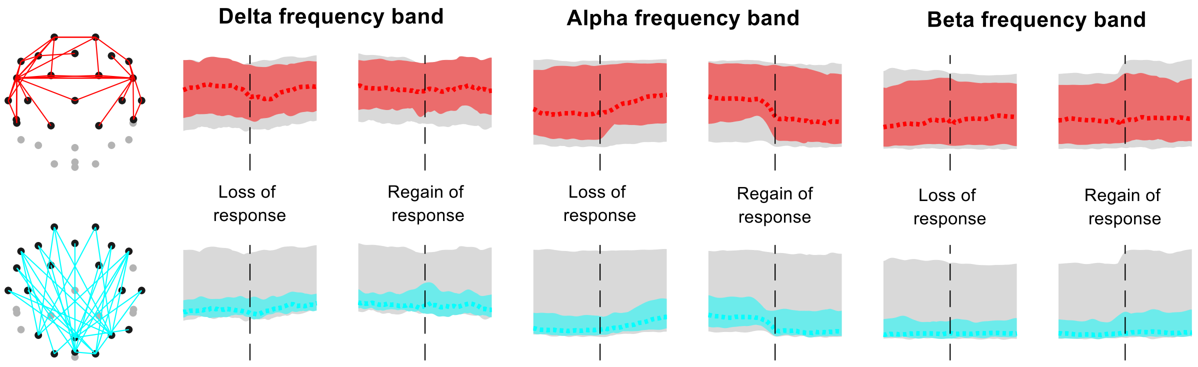

Supplement: Supplementary file 1 [file aln-133-774-s001.pdf]
